# The Duo of Osteogenic and Angiogenic Differentiation in ADSC-Derived Spheroids

**DOI:** 10.3389/fcell.2021.572727

**Published:** 2021-04-09

**Authors:** Anastasiya A. Gorkun, Daria P. Revokatova, Irina M. Zurina, Denis A. Nikishin, Polina Y. Bikmulina, Peter S. Timashev, Anastasiya I. Shpichka, Nastasia V. Kosheleva, Tamara D. Kolokoltsova, Irina N. Saburina

**Affiliations:** ^1^FSBSI Institute of General Pathology and Pathophysiology, Moscow, Russia; ^2^Institute for Regenerative Medicine, Sechenov University, Moscow, Russia; ^3^Faculty of Biology, Lomonosov Moscow State University, Moscow, Russia; ^4^Koltzov Institute of Developmental Biology of Russian Academy of Sciences, Moscow, Russia; ^5^World-Class Research Center “Digital Biodesign and Personalized Healthcare,” Sechenov University, Moscow, Russia; ^6^Chemistry Department, Lomonosov Moscow State University, Moscow, Russia; ^7^Department of Polymers and Composites, N.N.Semenov Federal Research Center for Chemical Physics, Russain Academy of Sciences, Moscow, Russia

**Keywords:** osteogenesis, agiogenesis, spheroid, 3D culture, tissue engineering, adipose-derived stromal cells (ADSCs)

## Abstract

Bone formation during embryogenesis is driven by interacting osteogenesis and angiogenesis with parallel endothelial differentiation. Thence, all *in vitro* bioengineering techniques are aimed at pre-vascularization of osteogenic bioequivalents to provide better regeneration outcomes upon transplantation. Due to appearance of cell–cell and cell–matrix interactions, 3D cultures of adipose-derived stromal cells (ADSCs) provide a favorable spatial context for the induction of different morphogenesis processes, including vasculo-, angio-, and osteogenesis and, therefore, allow modeling their communication *in vitro*. However, simultaneous induction of multidirectional cell differentiation in spheroids from multipotent mesenchymal stromal cells (MMSCs) was not considered earlier. Here we show that arranging ADSCs into spheroids allows rapid and spontaneous acquiring of markers of both osteo- and angiogenesis compared with 2D culture. We further showed that this multidirectional differentiation persists in time, but is not influenced by classical protocols for osteo- or angio-differentiation. At the same time, ADSC-spheroids retain similar morphology and microarchitecture in different culture conditions. These findings can contribute to a better understanding of the fundamental aspects of autonomous regulation of differentiation processes and their cross-talks in artificially created self-organizing multicellular structures. This, in turn, can find a wide range of applications in the field of tissue engineering and regeneration.

## Graphical Abstract

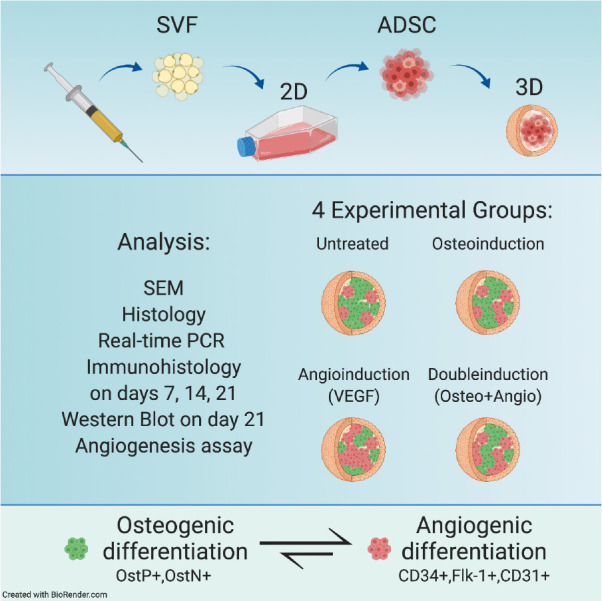

## Introduction

Numerous studies have shown that multipotent mesenchymal stromal cells (MMSCs) from various sources can supply factors supporting tissue regeneration into the local microenvironment. In particular, angiogenesis and osteogenesis can be stimulated through the secretion of specific cytokines, such as epidermal growth factor (EGF), basic fibroblast growth factor (bFGF), platelet-derived growth factor (PDGF), vascular endothelial growth factor (VEGF), keratinocyte growth factor (KGF), and angiopoietins (Chen et al., 2009; Langenbach et al., 2013; Yamaguchi et al., 2014). Clinical efficacy and availability of these cells have already been shown for treating bone defects at various locations (Corre et al., 2013).

It has long been recognized that osteogenesis and angiogenesis, including endothelial differentiation, are coupled and coordinated during bone formation (Schipani et al., 2009; Grosso et al., 2017). Therefore, it is crucial to create pre-vascularized structures since diffusion only allows osteoblasts to exchange gases and nutrients at distances not exceeding 200 μm (Muschler et al., 2004). In ideal tissue-engineered bone tissue constructions, functionality and structure, as well as regulation of both osteogenesis and vasculogenesis, should be combined, which could potentially solve the problem of obtaining massive artificial vascularized bone tissue fragments *in vitro*. Bioengineering is considered to be the most promising strategy for replacing or reconstructing large-scale defects (Song et al., 2016). According to the literature, there are different methods currently used to obtain vascularized bone tissue-engineered constructions (Laschke and Menger, 2012; Duttenhoefer et al., 2013; Roux et al., 2015). Different approaches were used to achieve vascularization: cells are immobilized on the substrate and co-cultured in monolayer or hydrogels (Annabi et al., 2014; Rohringer et al., 2014; Holnthoner et al., 2015).

One way to solve the problem of vasculogenesis in bone tissue is to use a combination of MMSCs with human umbilical vein endothelial cells (HUVEC) (Inglis et al., 2016; Strassburg et al., 2016) or peripheral blood cells (Holnthoner et al., 2015). Many groups work on the design and architecture of substrates or constructions, as well as the optimization of biologically active materials (Lovett et al., 2009; Sakaguchi et al., 2013; Holnthoner et al., 2015; Song et al., 2016). Combining biomaterials with 3D cultures is another possible strategy, e.g., a two-step digital light-processing technique for fabricating a bone-mimetic 3D hydrogel construct based on octacalcium phosphate (OCP), HUVEC spheroids, and gelatin methacrylate (GelMA) hydrogels (Anada et al., 2019).

Studies have shown that of different MMSC populations, adipose-derived stromal cells (ADSCs) have an advantage in terms of angio- and vasculogenesis since they contain not only a population of multipotent cells but also a vascular fraction that can contribute to the rapid restoration of blood supply to the damaged area (Marra and Rubin, 2012; Thery et al., 2015). Another unique feature of ADSCs is their ability to enhance angiogenesis through paracrine stimulation (Kalinina et al., 2015; Zhang et al., 2016). Altogether, this makes ADSCs a convenient cell source widely used in vascular regeneration-related studies for recreation of an angiogenic niche by using native (Cerino et al., 2017) as well as genetically modified ADSCs (Makarevich et al., 2015, 2018).

It is now widely accepted that 3D culturing of cells in the form of spheroids or organoids is a “bridge” between monolayer cultures and native tissues (Pampaloni et al., 2007). In 3D culture, cells acquire emergent functionality via multilevel interactions through cell junctions, microvesicle exchange, and extracellular matrix (ECM), which altogether provides the regulation of their collective behavior as well as restoration of initial cellular phenotype and functional activity (Repin et al., 2014; Zurina et al., 2018, 2020). Indeed, it was shown that such arrangements within spheroids as intercellular junctions and cell–matrix interactions result in the appearance of a local microenvironment, which facilitates proper cellular differentiation (Lee et al., 2016; Muller et al., 2019). In the case of ADSCs, cultivation in the form of spheroids allows, among other things, endothelial (Park et al., 2014; Gorkun et al., 2018; Saburina et al., 2018) and osteogenic differentiation (Gurumurthy et al., 2017; Saburina et al., 2018). However, simultaneous induction of multidirectional cell differentiation in MMSC-derived spheroids has not been considered earlier.

Thus, in this work, we aimed to explore the possibility of simultaneous induction of both angiogenic and osteogenic differentiation of ADSC employing spheroid culture system. The aim is to establish a platform where interactions between angiogenesis and osteogenesis can be easily manipulated and studied, employing high-throughput approaches.

## Materials and Methods

The study was conducted on the primary culture of human adipose-derived stromal cells (ADSCs). Adipose tissue fragments were collected from three donors (without chronic diseases, age 25–60) undergoing liposuction after receiving their written, informed consent. All the procedures were performed under local anesthesia and aseptic conditions and were approved by the Local Ethical Committee of Sechenov University (#07–17 from 13.09.2017, Moscow, Russia) while performed in accordance with the Helsinki Declaration.

The culture of human umbilical vein endothelial cells (HUVEC) was kindly provided by the “Collection of cell cultures for biotechnological and biomedical researches (of general biological and biomedical applications)” of the Koltzov Institute of Developmental Biology of Russian Academy of Sciences.

### 2D Culture of Human Adipose-Derived Stromal Cells

Biopsy samples were placed in a sterile transport container with DMEM/F-12 (BioLoT, Russia), L-glutamine (2 mM; BioLoT, Russia), and gentamicin (50 μg/ml; PanEco, Russia) and delivered to the laboratory.

Tissue samples were washed in Hank’s solution (BioLoT, Russia) containing antibiotics (1% penicillin–streptomycin, 400 U/ml of gentamicin; PanEco, Russia), minced mechanically, and digested with collagenase type I (0.07% solution) and dispase (0.025% solution) (PanEco, Russia) for 25 min at + 37°C. After incubation, DMEM/F-12 with 10% fetal clone serum (FCS; HyClone, United States) was added to the samples, and they were centrifuged for 5 min at 400 *g*. The pellet was resuspended in medium and filtered through a nylon filter to remove large tissue fragments. The cell suspension, together with small tissue fragments, was transferred to Petri dishes and cultured in complete growth medium consisting of DMEM/F-12 (1:1; BioLoT, Russia) supplemented with 2 mM L-glutamine, 100 U/ml of gentamicin (PanEco, Russia), 10% FCS (HyClone, United States), 1% 100 × ITS-G (insulin–transferrin–selenium) (BioLoT, Russia), 20 ng/ml of bFGF (ProSpec, Israel), and 15 U/ml of heparin (PanEco, Russia). ADSCs were cultured under standard conditions (37°C, 5% CO_2_). The medium was replaced every 3 days. As soon as cultures reached 90–95% confluence, the monolayer was treated with versene (BioLoT, Russia) and 0.25% trypsin solutions (BioLoT, Russia). The cells were passaged in a fresh portion of growth medium to new Petri dishes. Cell morphology and character of growth were controlled daily under an Olympus CKX-41 inverted phase-contrast microscope. Cells were cultured up to passage 4.

### Immunophenotyping of Human Adipose-Derived Stromal Cells in 2D Culture

The monolayer ADSC cultures at the fourth passage were immunophenotyped by the following surface marker proteins of multipotent mesenchymal stromal cells (Dominici et al., 2006; Haasters et al., 2009): CD105-PerCP-Cy5.5, CD90-FITC (BD Stemflow Positive cocktail, BD Bioscience, United States), CD73-PE, CD19-PE, CD29-PE, CD44-PE, CD31-PE (Miltenyi Biotec, Germany), CD45-PE, CD34-PE, CD14-PE, CD11b-PE, HLA-DR-PE (BD Stemflow Negative cocktail, BD Bioscience, United States), and CD146-PE (Biolegend, United States). Mouse isotypic controls IgG2a, IgG1 (Miltenyi Biotec, Germany), and unstained cells were used as controls.

Cells were washed from the complete growth medium with versene solution (BioLot, Russia), treated with 0.25% trypsin solution (BioLot, Russia), transferred to 15-ml tubes, and centrifuged (7 min, 400 *g*). The pellet was resuspended in PBS (pH 7.4) with 1% serum and incubated in the dark (15 min, 25°C) with antibodies (10 μl of antibodies per 1 × 10^6^ cells) conjugated with fluorescent labels [fluorescein isothiocyanate (FITC), phycoerythrin (PE), Cyanine 5.5 (Cy5.5)]. Stained cells were centrifuged (5 min, 400 *g*), and the pellet was resuspended in 1 ml of PBS containing 1% FCS in tubes for flow cytometry. The samples were analyzed on a SONY SH800 cell sorter (Sony biotechnology, United States).

To reveal the capacity for osteogenic and angiogenic cell differentiation, ADSC monolayers were incubated with osteoinductive medium and VEGF (see detailed protocol below) for 21 days. After day 21, cells were seeded on coverslips. Then coverslips were fixed in 4% PFA for 20 min at +4°C. Samples were then washed in cold PBS (PanEco, Russia) and stained with antibodies against osteopontin (Abcam, United Kingdom) and VEGFR2 Flk-1 (Thermo Scientific, United States) (see detailed protocol below).

### 3D Cultivation and Differentiation of Human Adipose-Derived Stromal Cells

Agarose multiwell plates were prepared from a 2% agarose solution (A-6013, Sigma-Aldrich, Germany) on a DMEM/F12 mixture (1:1) supplemented with 75 μg/ml gentamicin using 3D PetriDish molds (Microtissues^TM^, United States). Agarose plates were stored at +4°C in DMEM/F12 medium.

Cells at the fourth passage were treated with versene (BioLoT, Russia) and 0.25% trypsin (BioLoT, Russia) solutions to obtain cell suspension, transferred to 15-ml tubes, and centrifuged (7 min, 400 *g*). The resulting pellet was resuspended in the full growth medium to a concentration of 3.3 × 10^6^ cells/ml. Of the resulting suspension, 150 μl was transferred to non-adherent agarose plates. After an hour, 2 ml of complete growth or induction medium was added to the wells. Every microplate allowed us to collect up to 256 spheroids for further high-throughput analysis.

Spheroids were divided into four experimental groups: (1) Untreated spheroids with the regular growth medium; (2) Osteo group with the osteoinductive medium; (3) Angio group with the VEGF-supplemented growth medium; (4) Double group with the medium containing all components necessary for both osteo- and angiogenic differentiation.

For osteogenic differentiation, spheroids were cultivated in the complete growth medium supplemented with 100 nM dexamethasone (Sigma-Aldrich, Germany), 20 mM β-glycerophosphate (Sigma-Aldrich, Germany), and 0.05 mM ascorbic acid (Sigma-Aldrich, Germany).

Angiogenic differentiation was induced by adding 10 ng/ml of vascular endothelial growth factor VEGF (Sci-Store, Russia) to the complete growth medium. For double induction, all components necessary for both osteo- and angiogenic differentiation were added to the complete growth medium. The medium was replaced every 2 days. Cells in all groups were cultured under 3D conditions for 7, 14, and 21 days. For real-time PCR experiments, spheroids after 1 day in 3D culture were also included in the analysis.

### Immunocytochemical Staining of Spheroids From Human Adipose-Derived Stromal Cells

Immunocytochemical staining was performed on 3D cell cultures fixed with methanol for 12 h. Before staining, spheroids were washed from 100% methanol at room temperature sequentially in 80%, 60%, 40%, and 20% methanol solutions and in PBS for 10 min. Antigen retrieval procedure was used for CD31 staining: the samples were incubated with Tris-EDTA buffer (pH = 9) for 20 min at + 95°C. After washing, the samples were incubated in 250 μl of a solution of primary antibodies in PBS with 10% serum and 0.1% Tween-20 (24 h at +4°C). We used primary antibodies against vimentin (Abcam, R&D Systems, United States), CD31 (Thermo Scientific, United States), VEGFR2 Flk-1 (Thermo Scientific, United States), collagen type I (Abcam, United Kingdom), osteocalcin (R&D Systems, United States), and osteopontin (Abcam, United Kingdom). All antibodies were used at 1/500 dilution. Samples were then washed with PBS and incubated in 250 μl of a solution of secondary species-specific antibodies conjugated with fluorochromes Alexa Fluor 488 and Alexa Fluor 594 (ThermoScientific, United States). All antibodies were used at 1/500 dilution. The nuclei were stained with 2 μg/ml of intercalating dye Hoechst 33258 (Serva, Germany) or DAPI (Abcam, United Kingdom). The preparations in the mounting medium were covered with coverslips and examined under an Olympus Fluoview FV10I laser confocal scanning microscope (Olympus, Japan) in visible and UV light.

### Quantitative Analysis of Collagen and Vimentin Fiber Alignment

The fibers’ alignment and angles, stained with antibodies against vimentin and collagen type I, were measured on confocal images with the available software CurveAlign (UW–Madison^1^) in MatLab. Collagen type I and vimentin alignment measure the similarity of the orientations of cytoskeleton fibers in a defined area, calculated as the mean resultant vector length in circular statistics (Berens, 2009). The alignment coefficient ranged from 0.0 to 1.0, where higher alignment coefficients indicate fibers in a given image or regions of interest (ROI) to be more aligned. Measurement was performed using the curvelet fiber representation (CFR) mode of fiber analysis method (Liu et al., 2017). The diagrams representing angle distribution were built using Maple software.

### Scanning Electron Microscopy

For scanning electron microscopy (SEM), the samples were fixed with glutaraldehyde (3% solution in PBS) overnight at +4°C. Samples were then washed thrice with PBS and fixed in OsO_4_ (1% solution in PBS) for 1 h at room temperature. Then spheroids were washed thrice with PBS and dehydrated with ethanol (50% and 70%; twice for 5 min each). The samples were stored at +4°C in 70% ethanol. The samples were then further dehydrated with ethanol (80%—twice for 5 min; 96%—twice for 5 min) and acetone (5 min). Then we dried the samples at the critical point, covered them with golden particles in vacuum, and studied the replica using a CamScan-S2 scanning electron microscope (Cambridge Instruments, United Kingdom).

### Histology

Spheroids were collected and fixed in 4% PFA for 20 min at +4°C. Samples were then washed in cold PBS (PanEco, Russia), embedded in the HistoGel^TM^ (Richard-Allan Scientific^TM^, USAHG-4000-012), dehydrated in graded alcohol concentrations, and embedded in paraffin. Paraffin sections of 5-μm thickness were cut, deparaffinized, rehydrated, and stained with hematoxylin/eosin (H&E) (Sigma-Aldrich, Germany) to assess overall spheroids’ morphology.

### Analysis of Gene Expression by Real-Time Polymerase Chain Reaction

Spheroids were fixed in a 500-μl lysis mixture [1 ml of lysis buffer (Thermo Scientific, United States), 20 μl mercaptoethanol] and pipetted until spheroids were completely lysed. Samples were stored at −80°C.

GeneJET PCR purification kit (Thermo Scientific, United States) was used to isolate total RNA according to the manufacturer’s instructions. Then RNA was treated with DNase I to remove the genomic DNA. Samples were incubated overnight at −20°C in acetate, 0.5 μl of glycogen, and 96% ethanol to reprecipitate RNA, and then centrifuged for 20 min at 14,000 *g*. The supernatant was removed, and RNA concentration was measured using a Nanodrop 8000 spectrophotometer (Thermo Scientific, United States).

M-MLV reverse transcriptase (Evrogen, Russia) and random hexanucleotides (Evrogen, Russia) were used to synthesize cDNA. The obtained samples were used to the analyze gene expression listed in Table 1. For PCR reaction, commercial mixtures qPCRmix and SYBR + LowROX (Eurogen, Russia) were used. The sequences of the used primers are presented in Table 1. Synthetic oligonucleotides used as primers (Eurogen, Russia) were selected using PrimerSelect programs (DNA STAR, United States) based on sequential values obtained in the international NCBI database. Real-time PCR was performed using the 7500 Real-Time PCR Amplifier System (Applied Biosystems, United States); analysis of the results was performed in StepOne^TM^ and StepOnePlus^TM^ Software v2.3 (Applied Biosystems, United States). We used RPS18 (ribosomal protein) to normalize mRNA expression, which was specifically selected from eight commonly used genes for spheroids as the most stable. The selection was performed based on absolute quantification of reference gene expression for days 1, 7, 14, and 21 as in the protocol described previously (Nikishin et al., 2018). The absence of primer dimers was evaluated using the melting curves.

**TABLE 1:** List of primers used in the research.

**Primers**	**Primer sequence (5′-3′)**	**Primer description**	**Length (nt)**	**Tm (°C)**
hCD31_For	TTG TCT CCC GCT GGT TTT G	hCD31	19	58.2
hCD31_Rev	ATT GGC ATT TGG GAC TTG AT		23	56.9
hCD34 _For	CCT TGC AAC ATC TCC CAC TAA A	hCD34	22	59.8
hCD34 _Rev	CCC TCT CCC CTG TCC TTC TT		20	61.5
hCol1a1_For	ACC GAG GCC TCC CAG AAC	hCol1a1	18	62.4
hCol1a1_Rev	GTG CAG CCA TCG ACA GTG AC		20	60.3
hFlk1_For	TGG TCA GGC AGC TCA CAG TC	hFlk1	20	61.2
hFlk1_Rev	CCG GTT CCC ATC CTT CAA TA		20	57.2
hOsx_For	CAC TGC CCC ACC CCT TAG	hOsx	18	60.7
hOsx_Rev	CTT CCC CAC CCA TTC TTC A		19	57.6
hRunx2_Fow	TGT CAT GGC GGG TAA CGA	hRunx2	18	58.7
hRunx2_Rev	TTG GGG AGG ATT TGT GAA GAC		21	60.4
hBmp2_For	ACG AGG TCC TGA GCG AGT TC	hBmp2	20	61.2
hBmp2_Rev	ACC TGA GTG CCT GCG ATA CA		20	59.6
hRPS18_For	ACGCCGCCGCTTGTG CT	hRPS18	17	71.0
hRPS18_Rev	AGTTCTCCCGCCCTC TTGGTGA	hRPS18	22	70.4

### 3D Angiogenesis Assay

To evaluate the spheroids’ ability to form a capillary-like network, they were encapsulated within PEGylated fibrin hydrogels as previously described (Gorkun et al., 2018). PEGylated fibrin hydrogel was prepared according to the previously developed protocol (Shpichka et al., 2020). Briefly, fibrinogen was covalently bonded with polyethylene glycol (PEG) using O,O′-bis[2-(N-succinimidyl-succinylamino)ethyl]polyethylene glycol (PEG-NHS; Sigma-Aldrich, Germany) at a molar ratio of 5:1 (PEG-NHS: fibrinogen). The reaction mixture was incubated at 37°C for 2 h. The spheroids suspension was distributed in fibrinogen solution, and then the thrombin solution was added (fibrinogen to thrombin ratio 1:1). This mixture immediately formed a gel.

Spheroids from all four groups were cultured in gels for 7 days, in complete growth medium supplemented with 10 ng/ml of VEGF, changed every 2 days. The process of tubule growth was monitored using a CKX41 inverted phase-contrast microscope (Olympus, Japan).

### Immunocytochemistry of Spheroids in Fibrin Gel

Spheroids in fibrin gel were fixed in 4% paraformaldehyde (+4°C, overnight), washed thrice in PBS, permeabilized by 0.2% Triton X-100 (in PBS) for 10 min, and blocked with 5% goat serum in PBS. Samples were then incubated (+4°C, overnight) in the mix of PBS+0.1% Tween-20+5% goat serum with primary antibodies against CD31 (ab119339; Abcam), fibronectin (MA5-11981; Abcam), CD34 (ab54208; Abcam), and vimentin (ab92547; Abcam). After three times washing with PBST (PBS+0,1% tween), samples were then incubated in 250 μl of a solution of secondary species-specific antibodies conjugated with fluorochromes Alexa Fluor 488 and Alexa Fluor 594 (ThermoScientific, United States). All antibodies were used at 1/500 dilution. The nuclei were stained with 2 μg/ml of intercalating dye Hoechst 33258 (Serva, Germany) or DAPI (Abcam). The preparations in the mounting medium were covered with coverslips and examined under LSM 880 laser confocal scanning microscope (ZEISS, Germany) in visible and UV light.

### Western Blot

Spheroids were homogenized in RIPA buffer with protease inhibitor cocktail. Protein concentration was measured by the BCA Pierce^TM^ BCA Protein Assay Kit (Thermo Scientific). Samples were denatured in Laemmli sample buffer consisting of 2% SDS, 10% glycerol, 5% mercaptoethanol, 62.5 mM Tris (pH 6.8), and 0.004% bromophenol blue. Proteins were separated by 10% sodium dodecyl sulfate polyacrylamide gel electrophoresis (SDS-PAGE) and then electrophoretically transferred onto Immuno-Blot^®^ PVDF Membrane (Bio-Rad). The membrane was washed with PBS buffer + Tween-20 and then blocked with 5% BSA for 1 h and incubated overnight at 4°C with primary monoclonal antibodies against CD31 (ab119339, Abcam), osteopontin (ab8448, Abcam), and ACTB (Sigma-Aldrich A5441; 1:5,000). After washing five times in PBST, the membranes were incubated for 1 h at room temperature with peroxidase-conjugated secondary polyclonal goat antibodies anti-rabbit IgG (Jackson ImmunoResearch Laboratories 111–035–144; 1:50,000) and anti-mouse IgG (Jackson ImmunoResearch Laboratories 115-035-003; 1:50,000). After washing five times in PBST, conjugated antibodies were visualized using enhanced chemiluminescence in 0.1 M Tris–HCl, pH 8.5, 12.5 mM luminol, 2 mM coumaric acid, and 0.09% H_2_O_2_.

Expression levels were compared with HSP90 expression, since its expression remains stable both at the transcriptional level, as well as at the level of protein synthesis (Nikishin et al., 2018).

### Statistics

Statistical analysis of fibril alignment and PCR results was performed, and graphs were created using the Prism 8.0 GraphPad software package. The significant difference of data was confirmed via two- and three-way analysis of variance (ANOVA), Tukey’s test, and paired samples *t*-test for the *p*-value less than 0.05. At least five measurements of three independent samples (from three different donors) of each kind were collected; data are reported as the means ± SEM.

## Results

### Verifying Adipose-Derived Stromal Cells as Those Showing Specific Characteristics of Multipotent Mesenchymal Stromal Cell

**FIGURE 1:**
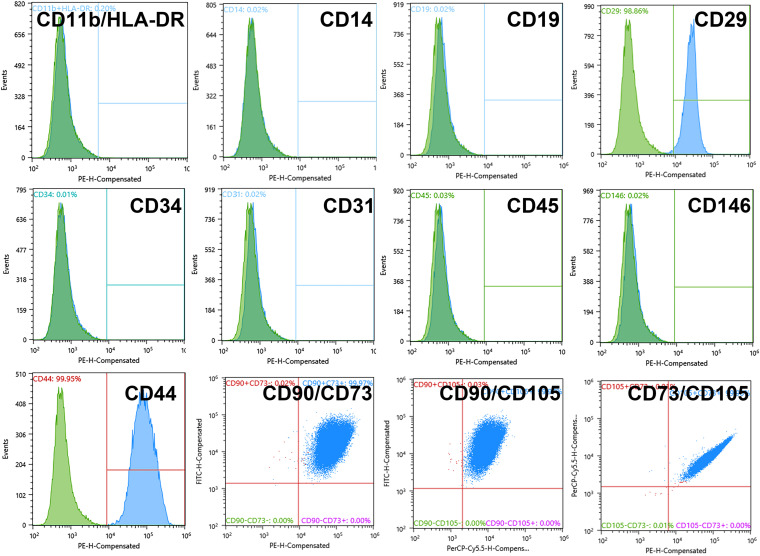
Adipose-derived stromal cell (ADSC) 2D culture showed a standard multipotent mesenchymal stromal cell (MMSC) phenotype at Passage 4 (before 3D cultivation). Negative marker levels: 0.02% of CD11b/HLA-DR cells, 0.02% of CD14 cells 0.02% of CD19 cells, 0.03% of CD45 cells, 0.01% of CD34 cells, 0.02% of CD31 cells, 0.02% of CD146 cells; positive marker levels: 98.86% of CD29 cells, 99.95% of CD44 cells, 91.94% of CD90 cells, 98.15% of CD105 cells, 99.99% of CD73 cells. The blue-filled histograms indicate the positively stained cells, while the green-filled histograms indicate the isotype-matched antibody controls. *Flow cytometry.*

Upon expansion in culture for four passages, the ADSCs displayed surface markers specific for the multipotent mesenchymal stem cells including CD29 (98.86 ± 0.07%), CD44 (99.95 ± 0.45%), CD105 (98.15 ± 0.91%), CD90 (91.94 ± 4.02%), CD73 (99.99 ± 0.01%) although not CD146 (0.02 ± 0,01%) (see also Figure 1). Double and triple multiparametric analysis showed 99.97 ± 3.57% of double CD73/CD90 cells, 99.97 ± 3.16% of CD90/CD105 cells, and 99.98 ± 0.82% CD73/CD105 cells. At the same time, the levels of markers for endothelial cells, endothelial progenitors, and blood cells (HLA-DR, CD11b, CD14, CD19, CD34, CD31, and CD45) were approaching zero (Figure 1) indicating that there were no such cells in the culture.

### ADSCs Are Capable of Dual Spontaneous Osteo- and Angiogenic Differentiation in 3D but Not 2D Culture

Next, we set to explore the possibility to promote simultaneous differentiation of these cells toward osteogenic and angiogenic directions.

**FIGURE 2:**
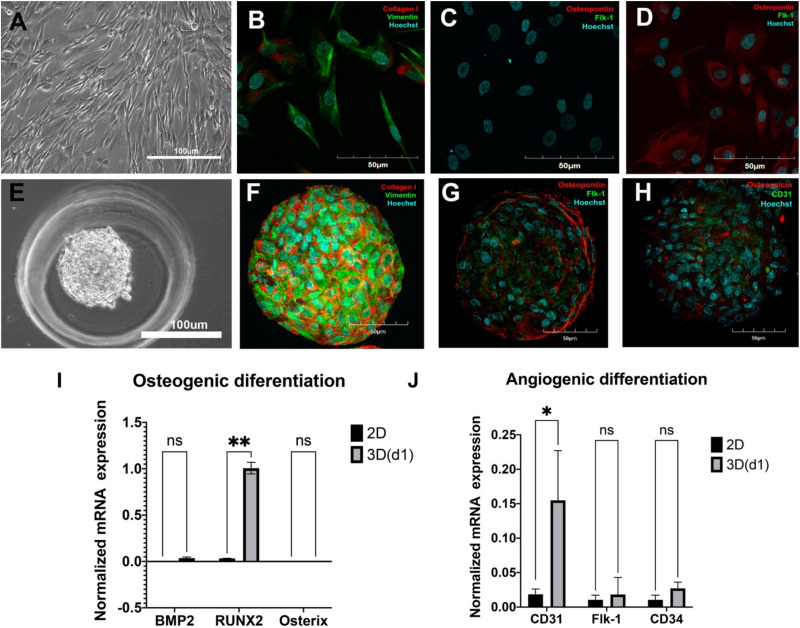
Spontaneous osteogenic and angiogenic differentiation of adipose-derived stromal cells (ADSCs) in 3D but not in 2D culture. **(A)** Cells were elongated and spindle shaped with a wave pattern of spreading. *Light Phase-Contrast Microscopy, scale bar 100* μ*m*. **(B)** ADSCs expressed vimentin (green) and collagen type I (red) in 2D culture. **(C)** Monolayer ADSC culture did not express osteopontin (red)/Flk-1(green). **(D)** After being cultured in the presence of osteogenic and angiogenic inducers, ADSCs in monolayer cultures started expressing osteopontin (red) but not Flk-1 (green). **(B–D)** The nuclei were counterstained with Hoechst 33258 (blue). *Laser Scanning Confocal Microscopy, scale bars 50* μ*m*. **(E)** ADSC aggregated into compact spheroid on day 1. *Light Phase-Contrast Microscopy, scale bar 100* μ*m*. **(F)** ADSCs expressed vimentin (green) and collagen type I (red) in 3D culture. **(G)** ADSC spheroid expressed osteopontin (red)/Flk-1(green) on day 7. **(H)** ADSC spheroid expressed osteocalcin (red) and CD31(green) on day 7. **(F–H)** The nuclei were counterstained with Hoechst 33258 (blue). *Laser Scanning Confocal Microscopy, scale bars 50* μ*m*. **(I)** Mean (± SEM) osteogenic gene (*BMP2*, *Runx2*, *Osterix*) expression levels (normalized to housekeeping genes) for 2D and 3D (day 1) cultures. **(J)** Mean (± SEM) angiogenic gene (*CD31*, *Flk-1*, *CD34*) expression levels (normalized to housekeeping genes) for 2D and 3D (day 1) cultures. Statistical analysis was performed by paired samples t-test, ^∗^*p* < 0.05, ^∗∗^*p* < 0.01.

Morphologically, ADSCs presented a homogeneous cellular population highly adhesive to the plastic, actively proliferating with spindle-shaped morphology (Figure 2A) and expression of collagen type I and vimentin as detected by immunohistochemistry (Figure 2B). Altogether with the described phenotype (see Figure 1), these features strongly indicate that the obtained ADSCs satisfy the classical characteristics of MMSCs.

At normal 2D culture conditions, ADSCs did not show any specific markers of osteoblasts (osteopontin) or endothelial cells (Flk1) (Figure 2C; see also FACS data for CD146, CD31, and CD34 in Figure 1). A combination of classical osteoinductive medium (see section “MATERIALS AND METHODS” for details) with VEGF (angioinductive agent) caused elevation of osteopontin, but not Flk-1 (Figure 2D). Arrangements of ADSCs into compact 3D spheroids (Figure 2E) immediately caused spontaneous and strong upregulation osteogenic markers osteopontin and osteocalcin as well as endothelial markers Flk-1 and CD31 (Figures 2G,H) and maintained expression of collagen I and vimentin on day 7 (Figure 2F).

However, RT-PCR revealed upregulation of osteogenic marker Runx2, but no BMP2, Osterix, (Figure 2I, qRT-PCR: 2D, 1 day in 3D), as well as angiogenic marker CD31, but not CD34 and Flk1 (Figure 2J, qRT-PCR) on day 1 in 3D culture.

Thus, these data indicate that arranging ADSCs in spheroids abruptly promotes expression of both osteo- and angiogenic markers compared with 2D cultures, thereby indicating a capacity for spontaneous dual osteo- and angiogenic differentiation in 3D.

### Adipose-Derived Stromal Cell Spheroids Subjected to Osteo- and Angio-Differentiation Maintain a Consistent Phenotype

**FIGURE 3:**
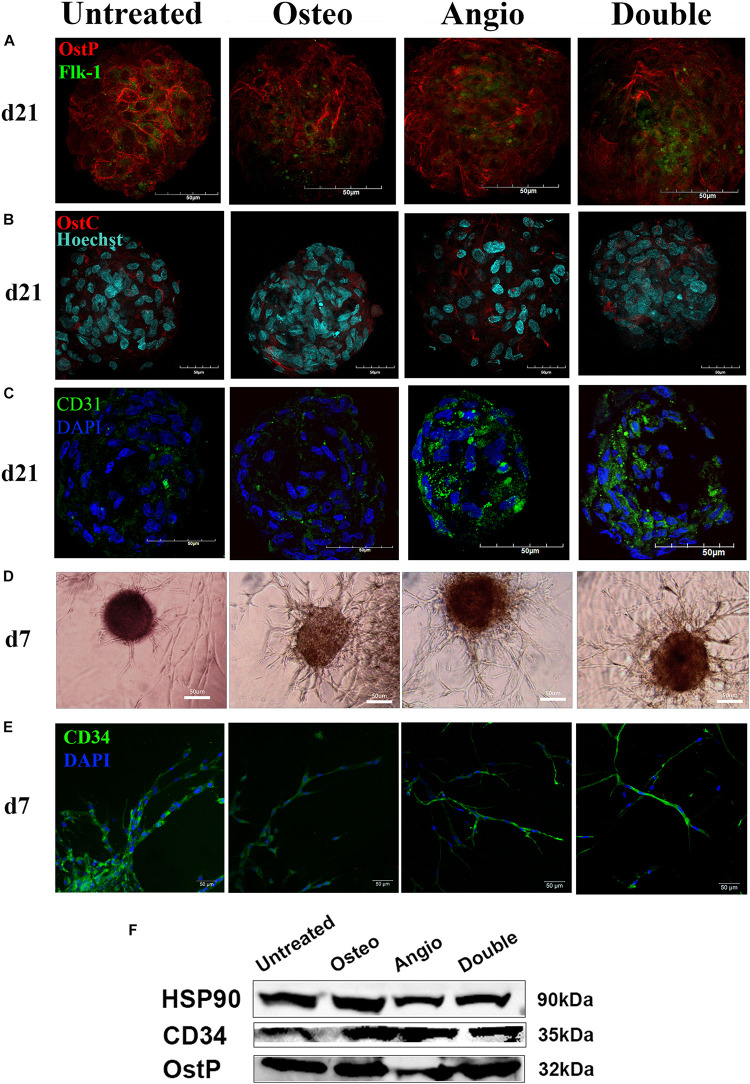
ADSC-derived spheroids showed similar expression of osteogenic (osteopontin, osteocalcin) and endothelial markers (Flk-1, CD31, CD34) with a similar for all groups capacity of angiogenesis in PEGylated fibrin gel. **(A)** The similar distribution of OstP (red) and Flk-1 (green) in spheroids for all groups on day 21. **(B)** The similar distribution of OstC (red) in spheroids for all groups on day 21. **(C)** Spheroids showed expression of PECAM1 (CD31) in all groups on day 21. **(D)** All groups showed an ability to form tubule-like structures in fibrin gels on day 7. **(E)** CD34-positive cells migrated from spheroids through fibrin gel and formed tubules on day 7. *Laser Scanning Confocal Microscopy, scale bar 50* μ*m*. *Light Phase-Contrast Microscopy, scale bar 50* μ*m.* **(F)** Similar expression of osteopontin (OstP) and CD34 compared with the housekeeping protein HSP90 in all groups on day 21 (blots were cropped and presented).

We further set to assess if this duo differentiation capacity is regulated by standard differentiation protocols toward osteogenic lineage or angiogenic lineage. For this purpose, the spheroids were cultivated in the presence of either standard osteogenic differentiation medium (dexamethasone, b-glycerophosphate, and ascorbic acid; called below *Osteo* group), standard angiogenic inductor VEGF (*Angio* group), or a combination of both (*Double* group). We matured spheroids in culture for 21 days and followed specific marker expression dynamics over this period. A clear trend was observed toward neither osteogenic nor angiogenic differentiation with time (Figure 3).

The immunocytochemical analysis of these spheroids revealed a high level of expression of an early marker of osteogenesis–osteopontin (OstP) in all groups (Figure 3A). At the same time, expression of osteocalcin (OstC), a late osteogenesis marker, was not as abundant as of OstP but present in all groups (Figure 3B). The markers of early endothelial cell differentiation (Flk-1) and mature endothelial cells (CD31) were more abundant in Angio and Double groups (Figures 3A,C). Angiogenesis assay demonstrated that spheroids from all groups were able to grow tubule-like structures in PEGylated fibrin gel in the presence of VEGF within 7 days (Figure 3D), and the cells forming these tubule-like structures were positive for CD34 (Figure 3E). Western blot analysis revealed a similar amount of osteopontin (OstP) and CD34 in all groups as housekeeping protein HSP90 (Figure 3F).

**FIGURE 4:**
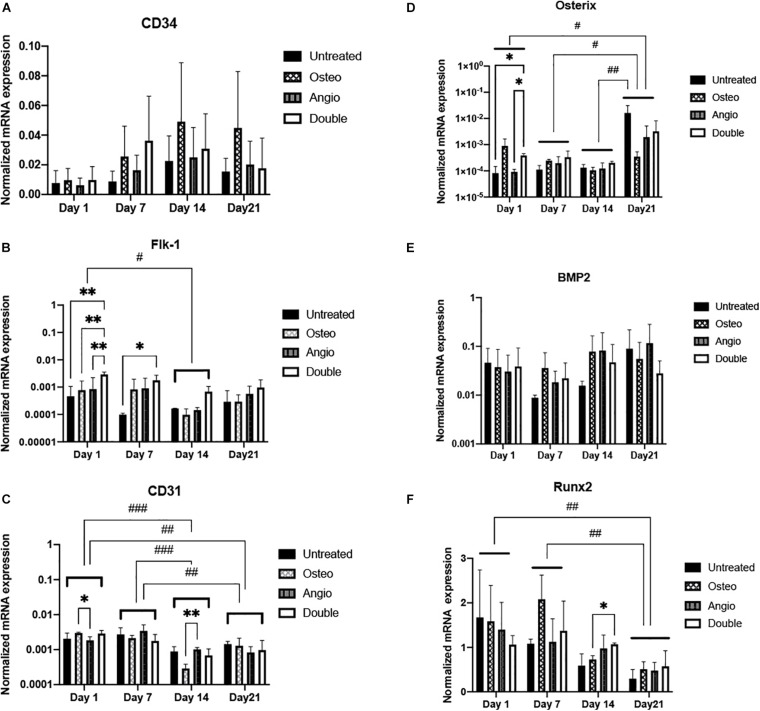
All groups of ADSC-derived spheroids showed different dynamics of gene expression in angiogenic and osteogenic differentiation. **(A)** Expression of *CD34* gene (early angiogenic differentiation marker) showed non-significant increase in all groups toward day 21. **(B)** *Flk-1* (VEGF receptor 2 gene) expression decreased significantly in all groups on day 14 compared with that on day 1. **(C)** *CD31* (mature endothelial cell marker) gene expression was significantly downregulated on days 14 and 21 compared with days 1 and 7. **(D)** *Osterix* marker showed a significantly increasing gene expression dynamics toward day 21. **(E)** All groups maintained consistent *BMP2* gene expression. **(F)** *Runx2* gene expression drastically regressed during 3D cultivation. Histograms represent mean (± SEM) gene expression levels (normalized to housekeeping genes). Two-way ANOVA describes significant differences between groups of the same time point (^∗^*p* < 0.05, ^∗∗^*p* < 0.01). Three-way ANOVA describes significant differences between time points (#*p* < 0.05, ##*p* < 0.01, ###*p* < 0.001).

These observations suggest that osteogenic differentiation within spheroids is rather spontaneous and independent on the presence of dexamethasone, b-glycerophosphate, and ascorbic acid. At the same time, angiogenic differentiation may be promoted in the presence of VEGF. To further elaborate on these observations, we have performed quantification of marker gene expression in spheroids using real-time PCR. Analysis of angiogenic markers (CD34, CD31, Flk1) did not reveal a time-dependent trend, elevation of angiogenic markers in either Angio or Double groups (Figures 4A–C). Similarly, expression of osteogenic markers (BMP2, Osterix, Runx2) did not demonstrate any time-dependent trend, but rather temporal fluctuations, e.g., elevation of osterix in Osteo group on day 7, but not on days 14 or 21 of culture (Figures 4D–F).

**FIGURE 5:**
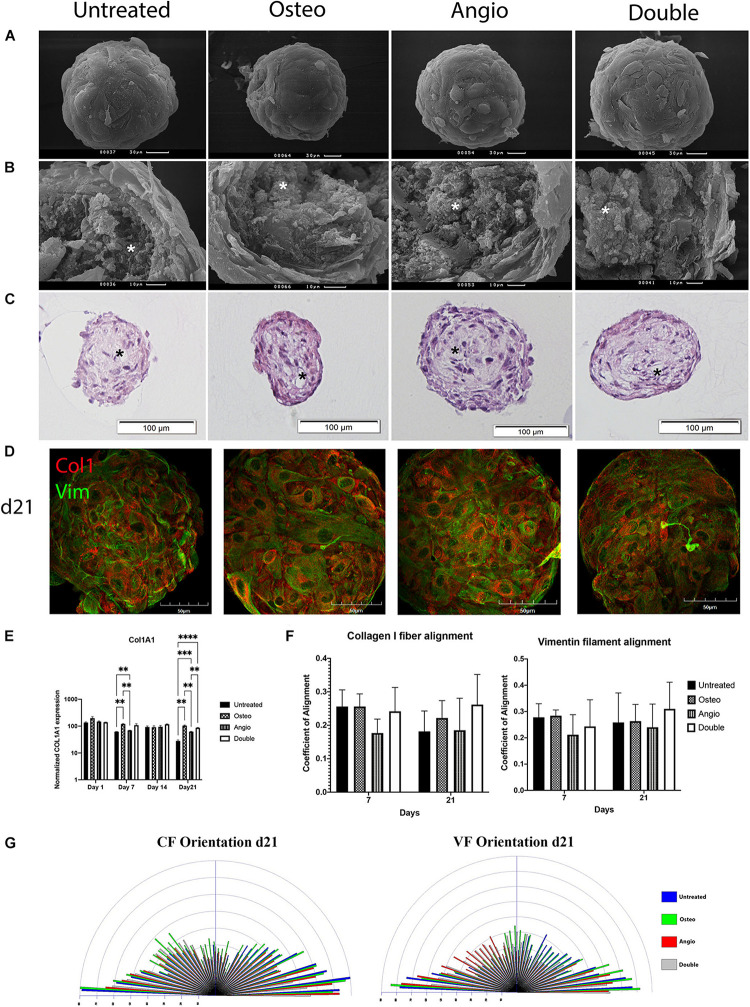
Untreated and treated ADSC-derived spheroids developed identical morphology by day 21 in 3D culture. **(A)** ADSCs aggregated in compact spheroids with a smooth surface formed by hexagonal-shaped cells; scale bar 30 μm. **(B)** Cracked spheroids revealed the layered structure of the surface and the central core composed of fibrils and bundles of extracellular matrix labeled by white asterisks; scale bar 10 μm. *Scanning electron microscopy*. **(C)** Histological H&E staining confirms that the surface of spheroids was formed by a few layers of cells and shows that the core had cavities labeled by black asterisks; scale bar 100 μm. *Bright-field microscopy*. **(D)** The distribution of Col1 (red) and Vim (green) in spheroids on day 21. *Laser Scanning Confocal Microscopy, scale bar 50* μ*m*. **(E)** Mean (± SEM) *COL1A1* gene expression levels (normalized to housekeeping genes), two-way ANOVA, ^∗∗^*p* < 0.01, ^∗∗∗^*p* < 0.001, ^*⁣*⁣**^p < 0.0001. **(F)** Col1 fibrils and Vim filament alignment coefficients. **(G)** The distribution and frequency of angles of collagen type I fibrils and vimentin filament orientation.

During this maturation process, the spheroids acquired a very smooth surface composed of a few layers of hexagonal-shaped cells tightly attached to each other (Figures 5A–C), whereas the central core had solitary scattered cells surrounded by abundant extracellular matrix including collagen type I (Figure 5D).

In all groups for all donors tested, ADSC spheroids maintained identical, external, and internal morphology with electron microscopy (Figures 5A,B) as well as histology with hematoxylin and eosin staining of their sections (Figure 5C). High expression levels of collagen type I (Col1) and vimentin (Vim) were noticed in all the groups (Figure 5D), and the levels of collagen type I (gene *COL1A1*) were further verified by RT-PCR (Figure 5E). There was mild but significant increase in the level of *COL1A1* on day 7 and day 21 of culture in both Osteo and Double groups compared with the untreated control (Figure 5E). We next explored alignment of collagen fibrils (CF) and vimentin filaments (VF), which revealed a general stability of random orientation indicated by a low coefficient (< 0.4) of anisotropy in all groups (Figure 5F). Angle’s frequency analysis showed a similar distribution of fibril orientation with rare significant differences between groups (Figure 5G).

These data stay in line with immunohistochemical and PCR analysis and altogether suggest that despite double osteo- and angiogenic differentiation, this occurs in 3D cultures spontaneously and is not regulated by standard protocols developed for 2D cultures.

## Discussion

We showed that adipose-derived stromal cells (ADSCs) with standard characteristics of multipotent mesenchymal stromal cells (MMSC) form spheroids of similar morphology and microarchitecture in different culture conditions creating microenvironment favorable for spontaneous and induced multidirection cell differentiation.

Adipose derived stromal cells belong to the broad family of MMSCs characterized by spindle-shaped morphology, molecular heterogeneity, multilineage differentiation, and secretion of numerous immunomodulatory and cytokine factors (Pittenger et al., 2019). ADSCs obtained from different donors satisfied the standard criteria of MMSCs (Hass et al., 2011) and showed the absence of endothelial cells, endothelial progenitors, blood cells, or osteoblasts in 2D culture. Additionally, as classic MMSCs, ADSCs showed a positive response to osteoinductive stimuli resulting in osteopontin expression and did not respond to angiogenic factors in monolayer. Herein, cells from different donors expressed collagen type I and vimentin, which have been previously shown to be typical for MMSCs from different sources (Konno et al., 2013; Amable et al., 2014).

When cultured in 3D, the situation changes drastically and assembling ADSCs into spheroids results in rapid and spontaneous upregulation of markers specific for both osteogenic and angiogenic differentiation. We suspect that spontaneous dual differentiation may be facilitated by the novel characteristics of cells’ microenvironment, which may, in turn, lead to changes in epigenetic landscape and/or increased stemness of MMSCs (Jauković et al., 2020). Indeed, several studies showed that cells in MMSC-derived spheroids, regardless of the source tissue, undergo spontaneous reprogramming including upregulation of pluripotent factors such as Sox2, NANOG, Oct4, and TERT (Cheng et al., 2012; Guo et al., 2014; Zhou et al., 2017). It was also shown that the differentiation process starts earlier and involves more cells in spheroids than in monolayer culture, which has been shown for osteogenic (Guo et al., 2014) and adipogenic (Wang et al., 2009) differentiation. Whether this is attributed to changes in cell epigenetic/stemness remains to be elucidated.

ADSC-derived spheroids maintained a similar structure under various differentiation conditions with the layered surface and the ECM-enriched core. This is consistent with the previously described morphology for MMSC-derived spheroids (Bellotti et al., 2016; Cesarz and Tamama, 2016; Kosheleva et al., 2017). Undoubtedly, these data imply morphogenesis going on in a similar way for all groups, including the untreated group.

The consistency of morphological processes was also confirmed by similar high expression and orientation of collagen type I fibrils and vimentin filaments (Figure 5). Their low degree of alignment (coefficient of anisotropy < 0.4) and distribution characterized non-mature tissue with the absence of mineralization in ADSC-derived spheroids and their high elastic properties (Georgiadis et al., 2016), which accorded primarily transverse orientation that is specific for Type I osteon (Bromage et al., 2003).

These results are matched with previous findings that MMSC-derived spheroids are characterized by increased ECM synthesis and changes in cytoskeleton organization and cell polarity. These novel mechanical properties provide a dynamic spatial context of cell–matrix and cell–cell interactions and lead to a significant rearrangement of physical forces acting on each cell within the 3D spheroid. Enhanced ECM secretion also provides a favorable environment for local growth factor and cytokine enrichment, supporting autocrine signaling (Bartosh et al., 2010).

Based on the described patterns of gene expression, the untreated group revealed spontaneous osteogenic and endothelial differentiation. However, most osteogenic and endothelial genes’ expression showed a lower trend in the untreated group compared with the groups with induction, which indicates the influence of VEGF and osteoinductive factors. On the other hand, patterns of gene expression dynamics during 3D cultivation were consistent among all groups. Immunohistochemical analysis of osteopontin, osteocalcin, CD31, and Flk-1 expression in all groups confirmed spontaneous osteogenesis and angiogenesis in ADSC spheroids (Figure 3). In general, the levels of expression of osteogenic markers significantly exceeded the levels of angiogenic marker expression that indicates predominance of osteogenic differentiation, which is consistent with previously observed MMSC’s potency to osteogenesis in spheroid (Imamura et al., 2020). Angiogenesis by CD34^+^ cells observed in all groups in PEGylated fibrin gel is confirming this observation and stays in line with the earlier study showing appearance of CD34^+^ cells in ADSC-derived spheroids (Bellagamba et al., 2018).

At the same time, we could not facilitate consistent either osteo- or angiogenic differentiation utilizing standard differentiation conditions. This can be attributed to the need for distinct differentiation protocols for 3D cultures. Alternatively, the complex interactions between various cell sub-types or cell–matrix interactions counterbalance the induced differentiation processes. We believe that more specific and more direct approaches are needed to simultaneously modulate multidirectional differentiation. Indeed, it has been shown that VEGF affect not only angiogenesis but may also influence osteogenesis (Lattanzi and Bernardini, 2012). Similarly, glucocorticoids used for standard osteogenic differentiation are also known to influence angiogenesis (Chen et al., 2018).

## Conclusion

In this study, we show that 3D culture promotes spontaneous multidirectional differentiation of ADSCs. Specifically, adipose tissue-derived MMSC-like cells acquire a capacity for spontaneous osteogenesis and partial endothelial-like differentiation when cultured in the form of spheroids. At the same time, standard osteogenic and angiogenic exogenic factors were not capable of influencing cell differentiation within spheroids in a persistent and time-dependent manner. We also show that ADSC-derived spheroids maintain stable morphology in different cultural conditions.

Our results open new approaches for *in vitro* generation of bioequivalents of vascularized bone tissue fragments and allow rapid and high-throughput analysis of interactions between osteogenic and angiogenic differentiation lineages. This opens a wide range of possibilities in both fundamental and applied research in the area of bone tissue development and regeneration.

## Data Availability Statement

The original contributions presented in the study are included in the article/supplementary material, further inquiries can be directed to the corresponding author/s.

## Ethics Statement

Adipose tissue fragments were collected from the patients undergoing liposuction after receiving their written informed consent. All the procedures were performed under local anesthesia and aseptic conditions and were approved by the Local Ethical Committee of Sechenov University (#07–17 from 13.09.2017, Moscow, Russia) while performed in accordance with the Helsinki Declaration.

## Author Contributions

AG, IZ, and IS contributed to the conception and the design of the study. DR and IZ wrote the first draft of the manuscript. AG, DR, and IZ prepared the submitted version of the study. DR and IZ performed 2D and 3D cell cultivation and collected samples for further analysis. AG and PB accomplished the flow cytometry and immunocytochemical staining. DR and DN performed the PCR and WB of all samples. IZ and AS performed the angiogenesis assay. IZ and NK provided the SEM, confocal microscopy, and statistical analysis. TK, PT, and IS supervised the study and edited the manuscript. All authors contributed to the manuscript revision, read and approved the submitted version.

## Conflict of Interest

The authors declare that the research was conducted in the absence of any commercial or financial relationships that could be construed as a potential conflict of interest.

## Footnotes

1 http://loci.wisc.edu/software/curvealign
